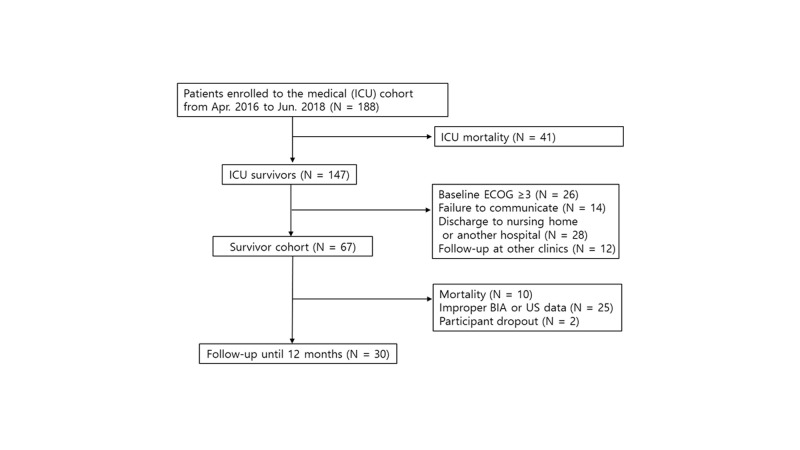# Association of baseline muscle mass with functional outcomes in intensive care unit survivors: A single-center retrospective cohort study in Korea

**DOI:** 10.1097/MD.0000000000039156

**Published:** 2024-08-09

**Authors:** Sung Yoon Lim, Jong Sun Park, Young-Jae Cho, Jae Ho Lee, Choon-Taek Lee, Yeon Joo Lee

**Affiliations:** a Department of Internal Medicine, Division of Pulmonology and Critical Care Medicine, Seoul National University College of Medicine, Seoul National University Bundang Hospital, Seoul, South Korea.

**Keywords:** bioelectrical impedance, intensive care units, physical fitness, skeletal muscle, survivors, ultrasonography

## Abstract

In critical care settings, ultrasound (US) of the quadriceps muscle and Bioelectrical Impedance Analysis (BIA) are noninvasive and widely available tools to evaluate muscle mass. We studied whether baseline muscle mass affects physical function in intensive care unit (ICU) survivors after discharge. This retrospective review of a prospective cohort enrolled 30 patients admitted to the medical ICU between April 2016 and June 2018. On ICU admission, quadriceps muscle thickness and skeletal muscle mass were measured using US and BIA, respectively. Muscle strength and physical function were measured using handgrip dynamometry, the 6-min walk test, and the Barthel index questionnaire survey during every clinic visit at 1, 3, 6, and 12 months after hospital discharge. Skeletal muscle mass at ICU admission was statistically correlated with the 6-min walk distance (6MWD) and Barthel index score. The segmental lean mass of the right arm was also positively correlated with handgrip muscle strength at 6 months after discharge. Likewise, the correlation between quadriceps muscle thickness at ICU admission and 6MWD at 6 months after discharge was positive and statistically significant. Multivariate regression analysis showed that skeletal muscle mass was associated with a reduced 6MWD, but the length of ICU stay was not. The segmental lean mass of the right arm also showed a significant association with handgrip strength after discharge. Low muscle mass on ICU admission is associated with reduced muscle strength, causing impaired physical function after hospital discharge in ICU survivors.

## 1. Introduction

Improved care of critically ill patients has decreased mortality rates, resulting in a large and expanding population of intensive care unit (ICU) survivors.^[1]^ Although many ICU survivors are discharged home after treatment, they are unable to perform their usual activities of daily living after hospital discharge.^[2]^ These patients have substantial morbidities that can persist from months to years after hospital discharge. Among those morbidities, impaired physical function that adversely affects daily function occurs in a majority of critical care survivors.^[3]^ Establishing a cohort of ICU survivors is essential not only for understanding the extent of these long-term effects but also for identifying the associated risk factors, which is crucial for developing targeted interventions and preventive strategies.

Numerous risk factors have been identified as contributing to the physical impairment observed in ICU survivors. Notably, the reduction of muscle mass during an ICU stay has emerged as a critical factor in the ensuing loss of physical function. Chan et al^[4]^ examined the lean muscle mass of survivors by using dual-energy X-ray absorptiometry at 6 and 12 months after acute respiratory distress syndrome. They found that rapid muscle wasting occurs during respiratory failure, leading to decreased whole-body lean mass in survivors. A greater percentage of lean mass was also significantly associated with gait speed and the 6-min walk distance (6MWD), but not with self-reported functional status.

The vulnerability of patients with low baseline muscle mass at the time of ICU admission to adverse outcomes has also been underscored. Previous studies have been published on the association of a decreased muscle mass at ICU admission with fewer ICU-free days and a higher in-hospital mortality in critically ill patients.^[5,6]^ Giani et al^[7]^ and Loosen et al^[8]^ have demonstrated the implications of low skeletal muscle mass as a determinant of adverse outcomes in critically ill patients. These outcomes range from increased mortality and ICU-acquired weakness to functional disabilities such as severe dysphagia.

However, the relation between lower skeletal muscle mass at ICU admission and long-term physical function after discharge remains unexplored. Given this gap, the current study hypothesizes that baseline muscle mass is significantly linked with impaired long-term physical function in ICU survivors. Both ultrasound (US) of the quadriceps muscle and bioelectrical impedance analysis (BIA) have been found to be useful tools for assessing skeletal muscle mass in critically ill patients and healthy individuals.^[9–12]^ Therefore, the objective of this study was to clarify the impact of low skeletal muscle mass assessed using BIA and US at ICU admission on long-term physical function in ICU survivors within a cohort population whose physical functions were systematically evaluated after discharge.

## 2. Methods

### 2.1. Study population

We conducted a retrospective analysis of data collected during an ongoing prospective cohort study at a tertiary academic hospital in Korea. Our medical ICU is equipped with 16 beds and staffed by 3 critical care physicians with a bed-to-nurse ratio ranging from 1:1.3 to 1:5. Patients 18 years of age or older who were admitted to the medical ICU for >24 hours because of respiratory failure between April 1, 2016, and June 30, 2018, were enrolled. The sample size for our study was inherently determined by the number of eligible patients admitted within this timeframe. This is typical of retrospective designs where the availability of records dictates the extent of analysis rather than prospective power calculations.^[13]^ The following patients were excluded: patients without mechanical ventilator support who were supposed to be discharged within 72 hours, patients admitted for minor procedures (cardiac catheterization, bronchoscopy, tracheostomy, etc), patients admitted for close observation after an elective operation, and patients who refused cohort enrollment. From this respiratory failure cohort, patients who survived hospital discharge and were assessed in the critical care outpatient clinic were registered as a “survivor subcohort.” Patients were excluded from the survivor subcohort if they had a baseline Eastern Cooperative Oncology Group performance score of ≥3 or could not communicate effectively owing to cognitive impairment before the ICU admission. Written informed consent was obtained from patients or their families. This study was performed according to the Helsinki Declaration and approved by the institutional review board of our hospital (approval no. B-1602/336-308) on March 7th, 2016.

## 3. Study design

**Figure 1.**
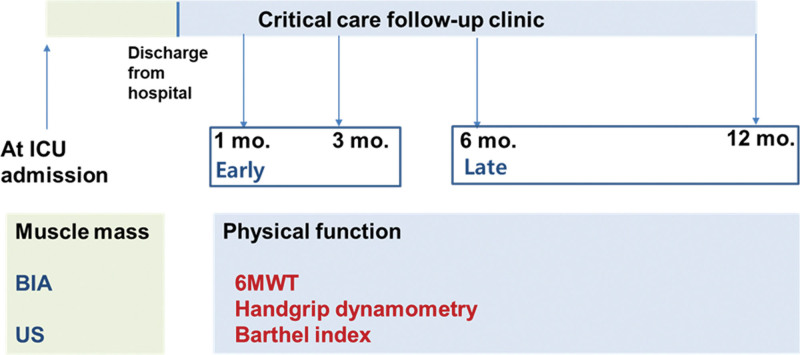
Study design for the survivor subcohort in the critical care follow-up clinic. 6MWT = 6-min walk test, BIA = bioelectrical impedance analysis, ICU = intensive care unit, mo = month, US = ultrasound (of the quadriceps muscle).

Every subject enrolled in the respiratory failure cohort underwent BIA and quadriceps US within 48 hours of admission to the ICU for measurements of baseline skeletal muscle mass and quadriceps muscle thickness, respectively. ICU survivors who were enrolled in the survivor subcohort were followed up until 12 months from the date of hospital discharge in the critical care outpatient clinic (Fig. 1). Muscle strength and physical function were measured using handgrip dynamometry, a 6-min walk test, and a Barthel index questionnaire survey during every clinic visit 1, 3, 6, and 12 months after hospital discharge. To target patients who may be capable of engaging in postdischarge interventions, participants were included in this analysis if they completed ≥1 physical assessment at 3, 6, or 12 months of follow-up.

## 4. Measurement of muscle mass

### 4.1. *Bioelectric impedance analysis*

Bioelectric impedance was measured with a portable device for segmental BIA (InBody S10®; Biospace, Seoul, South Korea), using 50-kHz alternating current. The InBody S10 body composition analyzer is designed for patients aged >3 years who are immobile or with amputation, is equipped with touch-type electrodes or adhesive-type electrodes, and produces results within 2 minutes. With each BIA test, whole-body and segmental (right and left arms, right and left legs, and trunk) fat, water, and muscle, including lean body mass, fat free mass and skeletal muscle mass were measured.

All measurements were performed within 48 hours of ICU admission; however, the time of measurement was not standardized. The patients underwent measurements in the supine position on a bed, with the arms and legs abducted from the body. Eight surface electrodes were placed on the thumb, middle fingers, and ball of the foot and heel. As the InBody S10 analyzer cannot measure height and body weight while the patient is lying down, height was measured in the supine position with a tape measure. We used the actual body weight of each patient, which was measured using a bed scale on the examination date in the ICU.

### 4.2. *Quadriceps muscle thickness*

The thickness of the quadriceps musculature was measured as described by Tillquist et al,^[14]^ by using a portable B-mode US device (General Electric Logiq e; GE Corp, Fairfield) equipped with a 7.5-MHz linear array transducer. The patients laid supine with both knees extended but relaxed and the toes pointing to the ceiling. The landmarks were set on the anterior surface of the quadriceps from the midpoint between the anterior superior iliac spine and the upper pole of the patella. Each midpoint was identified and marked on the skin with a surgical pen to ensure proper probe placement across repeated scans.

The transducer was oriented in the transverse and longitudinal planes perpendicular to the skin. The underlying tissues were then maximally compressed with the US probe to account for edematous effects on muscle thickness.^[8]^ For maximal compression, the investigators were instructed to apply as much pressure as the patient could tolerate. The distance between the upper margin of the femoral bone and the lower boundary of the deep fascia of the rectus femoris, incorporating both the rectus femoris and the vastus intermedius, was quantified using onscreen calipers. Each landmark was imaged 3 times and averaged across each leg and then between the 2 legs. Muscle mass measurements were conducted by 2 critical care physicians who had completed extensive training, including at least 30 supervised measurements, to ensure accuracy and uniformity. All measurements were carried out in a blinded manner to minimize any potential bias. For the initial 30 patients, measurements were performed simultaneously by both physicians to enhance the reliability of the results and to reduce interobserver variability. These concurrent measurements were overseen by a senior physician, providing an added layer of supervision to guarantee the precision of our data collection process.

## 5. Measurements of physical function

All physical function measurements were conducted by trained physical therapists who followed a standardized protocol in the outpatient setting.

### 5.1. *Six-minute walk test*

The 6-min walk test was conducted to assess overall physical function by using a single test and the longest walking distance possible. Previously published guidelines and procedures were followed,^[15]^ and the results were reported as the percentage of the predicted value based on established normative values.^[16]^

### 5.2. *Dynamometer measurements*

Handgrip strength was determined using the Jamar® Plus+ digital hand dynamometer (Patterson Medical, Warrenville). The reliability of the Jamar dynamometer has been confirmed in many populations.^[17]^ Three trials with a rest interval of 30 seconds between the tests were allowed for the dominant hand, and the patients were encouraged to exert their maximal grip strength. The best result from these 3 tests was used as the maximum handgrip strength.

### 5.3. *Barthel index questionnaire survey*

Functional evaluation included assessment of autonomy in basic activities of daily living, which were evaluated using the Barthel index.^[18]^ The Barthel index consists of quantitative scales ranging from 0 to 100, in which a score of 100 points denotes full autonomy in activities of daily living.

## 6. Data collection

The following data collected at ICU admission were extracted from the database of the prospective respiratory cohort: age, sex, admission category (medical or surgical), primary reason for ICU admission (pneumonia, heart failure, septic shock, and other), body mass index at study enrollment, Acute Physiology and Chronic Health Evaluation (APACHE) II score and Sequential Organ Failure Assessment (SOFA) score. Detailed information on missing rates is shown in Supplemental Table 1, http://links.lww.com/MD/N345.

## 7. Statistical analysis

Medians and proportions were used to describe the demographic and key clinical characteristics of the study population. Comparisons between paired groups were performed using the Wilcoxon signed-rank test. To evaluate the relationship between muscle mass parameters at ICU admission and the physical function after discharge, Pearson correlation coefficients were calculated separately for the muscle mass measured using US and BIA. To further investigate the associations between the 6MWD or handgrip strength (in absolute value) as the dependent variable and the various explanatory variables, we conducted a linear regression analysis. Variables that were statistically significant (*P* < .05) in the univariate analysis were included in the multivariate regression analysis with forward conditional elimination of data. Results were presented as coefficients with 95% confidence intervals. All analyses were performed using R software (version 3.3.2; http://www.R-project.org), and differences were considered statistically significant at *P* values of < .05.

## 8. Results

In total, 188 patients with respiratory failure were enrolled in the respiratory cohort and 147 patients (78.19%) were alive after ICU care. Of the 67 patients enrolled in the survivor subcohort, 25 had improper US or BIA data, 10 died during the following months after hospital discharge, and 2 withdrew for personal reasons unrelated to the study; thus, a total of 30 patients were followed up until 1 year (Supplemental Fig. 1, http://links.lww.com/MD/N347).

## 9. Baseline characteristics, muscle mass, and physical function of the study population

The study population had a median age of 75.0 years, and 85% were men. The median APACHE II and SOFA scores at ICU admission were 21.0 and 7.0, respectively. The other baseline characteristics included in this study are described in Table 1. The baseline skeletal muscle mass measured using BIA and the quadriceps muscle thickness measured using US at ICU admission in critical illness survivors are summarized in Supplemental Table 2, http://links.lww.com/MD/N346. Table 2 shows the muscle strength and physical function after ICU discharge, measured in critical care outpatient clinics. During the follow-up in outpatient clinics, the physical conditions of survivors showed significantly improved values in terms of the median 6MWD, handgrip strength, and Barthel index score.

**Table 1:** Baseline characteristics of intensive care unit survivors.

Characteristics	N = 30
Age (yr)	75.0 (67.0–78.0)
Sex (male, %)	25 (83.3)
BMI (kg/m^2^)	22.6 (21.0–27.8)
APACHE II score	21.0 (15.0–27.0)
SOFA score	7.0 (4.0–9.0)
Vasopressors n (%)	25 (83)
Neuromuscular blocker n (%)	9 (30)
Steroid n (%)	23 (77)
Charlson comorbidity score	
Comorbidities n (%)	4.0 (3.0–6.0)
Hypertension	12 (40)
Diabetes	4 (13)
COPD	6 (20)
Reason for ICU admission n (%)	
Pneumonia	25 (83.3)
Heart failure	1 (3.3)
Septic shock	2 (6.7)
Others	2 (6.7)
ARDS, n (%)	12 (40.0)
Mechanical ventilation, n (%)	25 (86.2)
CRRT, n (%)	26 (89.7)
Initial vital signs at ICU admission	
Mean artery pressure (mm Hg)	89 (79–99)
Pulse rate (beats/min)	105 (94–113)
Respiratory rate (breaths/min)	23 (20–27)
Temperature (°C)	36.75 (36.23–37.55)
Laboratory data on day of ICU admission	
pH	7.37 (7.27–7.43)
PaO_2_ level (mm Hg)	40 (35–50)
PaCO_2_ level (mm Hg)	91 (79–123)
White blood cell count (×10^3^/μL)	12 (9–18)
Hemoglobin level (g/dL)	10.55 (9.15–12.60)
Platelet count (×10^3^/μL)	182 (128–289)
Creatinine level (mg/dL)	1.27 (0.74–1.80)
Blood urea nitrogen level (mg/dL)	27 (20–35)
Total Protein (mg/dL)	5.90 (5.30–6.28)
Albumin (mg/dL)	2.65 (2.10–3.00)
Length of mechanical ventilation (d)	5.8 (3.0–6.8)
Length of ICU stay (d)	6.5 (4.0–9.0)
Length of hospital stay (d)	21.0 (14.0–37.0)

Values are expressed as median (interquartile range).

APACHE II = acute physiology and chronic health evaluation II, ARDS = acute respiratory distress syndrome, BMI = body mass index, COPD = chronic obstructive pulmonary disease, CRRT = continuous renal replacement therapy, ICU = intensive care unit, SOFA = Sequential Organ Failure Assessment.

**Table 2:** Muscle strength and physical function of intensive care unit survivors after hospital discharge.

Muscle strength and physical function	Early (1–3 mo)	Late (6–12 mo)	*P* value
Six-minute walk distance			
Measurement (m)	322.0 (206.0–400.0)	426.4 (349.4–494.0)	.032
% predicted	60.78 (25.94–69.31)	86.81 (64.09–95.17)	.001
Handgrip strength*			
Measurement (kg)	22.4 (16.6–29.6)	27.9 (10.5–31.3)	.045
% predicted	72.82 (56.4–90.0)	83.34 (79.38–93.57)	.005
Barthel index	91.7 (72.5–98.3)	100.0 (97.5–100.0)	.037

Values are expressed as median (interquartile range).

* Handgrip strength results are reported as the maximum force generated on the dominant side.

## 10. Correlation between muscle mass and physical function

To investigate the correlation between muscle mass at ICU admission and physical function after ICU discharge, we calculated Pearson correlation coefficients. The skeletal muscle mass obtained from BIA was positively correlated with the 6MWD and Barthel index score. Additionally, a significant correlation was observed between the segmental lean mass of the right arm and handgrip muscle strength (Table 3). Likewise, the correlation between quadriceps muscle thickness at ICU admission and 6MWD at 6 months after discharge was positive and statistically significant. However, quadriceps muscle thickness was not statistically correlated with the handgrip muscle strength and Barthel index score (Table 3).

**Table 3:** Correlation between baseline muscle mass at intensive care unit admission and physical function after hospital discharge in survivors.

Muscle strength and physical function	Skeletal muscle mass	Segmental lean mass				Quadriceps muscle thickness		
		Right arm	Left arm	Right leg	Left leg	Right	Left	Mean
Six-minute walk distance								
Early (1–3 mo)	0.451 (0.031)*	0.326 (0.129)	0.293 (0.174)	0.333 (0.121)	0.304 (0.159)	0.294 (0.311)	0.463 (0.096)	0.383 (0.178)
Late (6–12 mo)	0.741 (0.002)*	0.367 (0.178)	0.420 0.119)	0.690 (0.004)*	0.742 (0.002)*	0.604 (0.086)	0.720 (0.029)*	0.658 (0.054)
Handgrip strength								
Early (1–3 mo)	0.372 (0.056)	0.478 (0.012)*	0.330 (0.093)	0.292 (0.139)	0.242 (0.223)	0.143 (0.400)	0.235 (0.606)	0.192 (0.492)
Late (6–12 mo)	0.415 (0.097)	0.531 (0.028)*	0.358 (0.071)	0.343 (0.178)	0.366 (0.149)	0.072 (0.847)	0.166 (0.646)	0.113 (0.752)
Barthel index								
Early (1–3 mo)	0.448 (0.017)*	0.231 (0.237)	0.274 (0.159)	0.471 (0.011)*	0.440 (0.019)*	0.095 (0.737)	0.076 (0.788)	0.088 (0.756)
Late (6–12 mo)	0.476 (0.020)*	0.216 (0.501)	0.229 (0.269)	0.494 0.010)*	0.442 (0.010)*	0.372 (0.287)	0.187 (0.604)	0.290 (0.415)

Values are expressed as correlation coefficient, *R* value (*P* value).

* *P* < .05.

## 11. Associations of muscle mass with physical function

To further investigate which parameters had a significant influence on physical function after discharge, a multiple regression analysis was performed (Table 4). The 6MWD significantly increased with increasing skeletal muscle mass, but not with increasing APACHE II score or length of ICU stay. The segmental lean mass of the right arm obtained from BIA showed a significant association with the handgrip strength (Table 4).

**Table 4:** Linear regression analysis for assessing the associations of baseline muscle mass and physical function after hospital discharge.

Factors associated with 6-min walk distance	*β* coefficient	*P* value
Age	−5.7780	.0459
APACHE II score	0.9778	.7984
Charlson comorbidity score	−10.4378	.4541
Skeletal muscle mass at ICU admission	16.3403	.0016
Length of ICU stay	6.6356	.1056

Factors associated with Handgrip strength	*β* coefficient	*P* value
Age	−0.1178	.4585
APACHE II score	0.0269	.8961
Charlson comorbidity score	0.1848	.8018
Segmental lean (RA) at ICU admission	7.3694	.0117
Segmental lean (LA) at ICU admission	4.8475	.0929
Length of ICU stay	−0.0068	.9788

β coefficients represent the percentage change in the physical function variable per unit change in exposure variable.

APACHE II = acute physiology and chronic health evaluation II, CI = confidence interval, ICU = intensive care unit, LA = left arm, RA = right arm.

## 12. Discussion

In the present study, we found that muscle mass at ICU admission was significantly associated with physical function after discharge in ICU survivors. Baseline muscle mass was assessed using BIA or US at ICU admission, and physical function was evaluated using the 6-min walk test, handgrip dynamometry, and Barthel index questionnaire survey until 1 year after hospital discharge. The skeletal muscle mass obtained from BIA was highly correlated with the 6MWD, handgrip strength, and Barthel index score. Compared with the 6MWD at the first 3 months after discharge, the following period, up to 1 year, was more highly correlated with the skeletal muscle mass and segmental lean mass of both legs. As additional functional recovery was achieved in the late follow-up period compared with the early follow-up period, a stronger correlation was observed 3 months after discharge. In keeping with our results, Fan et al^[19]^ described that muscle weakness usually recovers within 12 months after discharge in acute respiratory distress syndrome survivors. In a study of previously healthy ICU survivors, maximal functional recovery was achieved in the first 3 to 6 months after discharge without additional improvement in the subsequent 6-month period.

Among the segmental lean mass measurements taken at ICU admission, the lean mass of the right arm was statistically correlated with handgrip strength measured with a dynamometer in the follow-up at critical care clinics. Because the dynamometer measurement was performed in the dominant hand (27 right-handed and 3 left-handed subjects), a correlation was observed between the lean mass of the right arm and the dynamometer-measured handgrip strength. Quadriceps muscle thickness assessed with the US at ICU admission was also statistically correlated with the 6MWD after ICU discharge. However, quadriceps muscle thickness was not correlated with handgrip strength measured with a dynamometer or with the Barthel index. Unlike skeletal muscle mass measured using BIA, quadriceps muscle thickness was only correlated with the 6MWD but not with the handgrip strength or Barthel index, indicating that quadriceps muscle thickness only reflects ambulatory function rather than whole-body physical function.

The relationship between skeletal muscle mass and 6MWD was retained even after adjustment for age, APACHE II score, and length of ICU stay. Both skeletal muscle mass and the segmental lean mass of the right arm were also associated with handgrip strength after adjustment for the same covariates. The length of ICU stay, a well-known risk factor for muscle wasting and weakness in patients admitted to the ICU,^[6,12]^ was not found to be a predictor of the 6MWD or handgrip strength in our multivariate regression analysis. Similarly, in the study of general ICU survivors, muscle strength did not correlate with the patients’ length of ICU or hospital stay, and the length of ICU stay was no longer statistically significantly correlated with muscle strength after adjustment.^[20]^

Similar to our results, several studies have reported a strong association between low muscle mass and limited physical function^[21]^ or objective physical performance^[22]^ in elderly patients. Janssen et al^[21]^ showed that there was an increased likelihood of functional impairment in older men and women with sarcopenia, as determined using the skeletal muscle index from BIA, relative to those with a normal muscle mass. These associations between sarcopenia and functional impairment remained significant after adjustment for age, race, body mass index, health behaviors, and comorbidity. Selva Raj et al^[23]^ also reported that leg muscle thickness assessed with the US was associated with functional performance and knee extensor strength in older adults.

A potential explanation is the positive legacy effect of baseline muscle mass on subsequent physical function in ICU survivors. Although muscle wasting occurs early and rapidly during the first week after admission to the ICU,^[24]^ the remnant baseline muscle mass after the initial wasting may be sustained until discharge. As muscle mass is known to be linearly correlated with muscle strength,^[25]^ the relationship between muscle mass and physical function has been suggested to be similar to the relationship between muscle strength and physical function.^[26]^ Thus, in this study, the initial muscle mass at admission impacted the physical performance after discharge.

Although muscle wasting during the ICU stay is a well-known contributor to poor physical function in survivors, we demonstrated that low baseline muscle mass was an additional risk factor for poor functional outcomes. A recent prospective observational study reported that more than two-thirds of patients who survive at least 1 year after ICU discharge have a similar functional autonomy (Barthel index) to that at baseline before ICU admission.^[18]^ Additionally, Weijs et al^[27]^ demonstrated that low muscle mass at admission in critically ill patients is associated with increased disability and an increased frequency of discharge to a nursing home.

In the present study, we assessed muscle mass using BIA or US rather than computed tomography (CT) or dual-energy X-ray absorptiometry. Transfer of critically ill patients for CT scans or dual-energy X-ray absorptiometry is not without risk and may be ethically difficult to justify for the purpose of muscle mass measurement. However, BIA and the US provide simple, inexpensive, and reliable estimates for this purpose. Unlike CT, bedside measurements of muscle mass are possible with existing portable ICU equipment such as BIA or US. In a recent study conducted on 135 critically ill patients, skeletal muscle mass measured using BIA showed a high correlation with and had greater values than skeletal muscle mass calculated from CT scans. In a study validating the use of US for muscle thickness measurements in critically ill patients, a moderate correlation was observed between quadriceps muscle thickness from US and abdominal muscle cross-sectional area from CT.^[10]^

The observed significant correlation between baseline muscle mass at ICU admission and subsequent physical function underscores its potential role in shaping targeted rehabilitation strategies for ICU survivors. By leveraging the baseline muscle mass measurements obtained via BIA and US, healthcare providers could tailor postdischarge care plans more precisely. Utilizing these accessible and noninvasive tools in the critical care setting may offer a pragmatic advantage, facilitating their integration into standard clinical evaluations. Such a personalized approach to rehabilitation could not only maximize the effectiveness of recovery interventions but also ensure that resources are allocated efficiently.

Our study has several strengths, including the prospective enrollment of patients, long-term follow-up, inclusion of only medical patients with a good baseline health status, and blinded assessment of body composition and quadriceps thickness to assess patient status objectively. On the other hand, a limitation of this study was the relatively small sample size with predominantly male patients, owing to the limited recruitment of patients who survived and visited the critical care follow-up clinic until 1 year, potentially creating a selection bias. This bias may skew our findings toward outcomes that are more typical for males or for individuals who can attend follow-up, potentially excluding those with more severe outcomes or those who face barriers to follow-up care. Although our sample is not very large, it is rather homogeneous and represents real-world data. Future studies would benefit from including more patients with a more balanced gender distribution. Additionally, the potential for inaccuracy in BIA and US measurements due to edema in critically ill patients presents a further limitation. Edema, common in this patient population, can distort the muscle mass measurement by increasing the fluid volume within the tissue, potentially leading to overestimating muscle mass. However, measurements were conducted relatively early, within 48 hours of ICU admission and before significant fluid overload could occur. The average ECW/TBW ratio for our study population was 0.4, situated at the upper limit of the normal range, which suggests minimal edema was present at the time of assessment. Furthermore, we acknowledge that measuring the muscle mass only at ICU admission and not during follow-up postdischarge visits could limit understanding of the muscle mass trajectory over time. Subsequent research incorporating BIA or US measurements at multiple time points postdischarge would be invaluable in providing a more dynamic picture of the functional outcomes in ICU survivors. Lastly, our patients were recruited from a single center; thus, a multicenter observational study is needed to validate our findings.

In conclusion, baseline muscle mass at ICU admission is associated with physical function after discharge in ICU survivors. In this regard, BIA or US muscle mass measurements could provide a practical tool for identifying individuals at an increased risk for functional impairment and physical disability after discharge. Increased awareness and meticulous care are required for patients with low muscle mass at ICU admission. Future research should investigate if changes in the post-ICU management of these patients could improve their physical outcomes.

## Author contributions

**Conceptualization:** Sung Yoon Lim, Jong Sun Park, Young-Jae Cho, Jae Ho Lee, Choon-Taek Lee, Yeon Joo Lee.

**Data curation:** Sung Yoon Lim, Jae Ho Lee, Yeon Joo Lee.

**Formal analysis:** Sung Yoon Lim, Yeon Joo Lee.

**Funding acquisition:** Choon-Taek Lee, Yeon Joo Lee.

**Investigation:** Young-Jae Cho, Yeon Joo Lee.

**Methodology:** Sung Yoon Lim, Jong Sun Park, Young-Jae Cho, Jae Ho Lee, Choon-Taek Lee, Yeon Joo Lee.

**Project administration:** Yeon Joo Lee.

**Resources:** Young-Jae Cho, Jae Ho Lee, Choon-Taek Lee, Yeon Joo Lee.

**Supervision:** Jong Sun Park, Jae Ho Lee, Choon-Taek Lee, Yeon Joo Lee.

**Validation:** Jong Sun Park.

**Writing – original draft:** Sung Yoon Lim, Yeon Joo Lee.

**Writing – review & editing:** Sung Yoon Lim, Jong Sun Park, Young-Jae Cho, Jae Ho Lee, Choon-Taek Lee, Yeon Joo Lee.

## Supplementary Material

**Figure:**