# Supplementary material for: Association of baseline muscle mass with functional outcomes in intensive care unit survivors: A single-center retrospective cohort study in Korea
Source: Medicine (Baltimore). 2024 Aug 9;103(32):e39156. doi: 10.1097/MD.0000000000039156 (PMC11315508; doi:10.1097/MD.0000000000039156)
Supplement: Supplementary file 1 [file medi-103-e39156-s001.docx]

Supplemental Table 1. Percentage of Missing Data

| Characteristics | N = 30 |
| --- | --- |
| Age | 0% |
| Sex | 0% |
| BMI | 0% |
| APACHE II score | 3.3% |
| SOFA score | 3.3% |
| Vasopressors | 0% |
| Neuromuscular Blocker | 0% |
| Steroid | 0% |
| Charlson comorbidity score  Comorbidities | 3.3% |
| Hypertension | 3.3% |
| Diabetes | 3.3% |
| COPD | 3.3% |
| Reason for ICU admission |  |
| Pneumonia | 0% |
| Heart failure | 0% |
| Septic shock | 0% |
| Others | 0% |
| ARDS | 0% |
| Mechanical ventilation | 0% |
| CRRT | 0% |
| Initial vital signs at ICU admission |  |
| Mean artery pressure | 0% |
| Pulse rate | 0% |
| Respiratory rate | 0% |
| Temperature | 0% |
| Laboratory data on day of ICU admission |  |
| pH | 0% |
| PaO2 level | 0% |
| PaCO2 level | 0% |
| White blood cell count | 0% |
| Hemoglobin level | 0% |
| Platelet count | 0% |
| Creatinine level | 0% |
| Blood urea nitrogen level | 0% |
| Total Protein | 0% |
| Albumin | 0% |

BMI: body mass index; APACHE II: Acute Physiology and Chronic Health Evaluation II; SOFA: Sequential Organ Failure Assessment; COPD: Chronic Obstructive Pulmonary Disease; ARDS: acute respiratory distress syndrome; CRRT: continuous renal replacement therapy; ICU: intensive care unit.
