# Supplementary material for: Association of baseline muscle mass with functional outcomes in intensive care unit survivors: A single-center retrospective cohort study in Korea
Source: Medicine (Baltimore). 2024 Aug 9;103(32):e39156. doi: 10.1097/MD.0000000000039156 (PMC11315508; doi:10.1097/MD.0000000000039156)
Supplement: Supplementary file 2 [file medi-103-e39156-s003.docx]

Supplemental Table 2. Baseline skeletal muscle mass and quadriceps muscle thickness at intensive care unit admission

|  | | Total (N = 30) |
| --- | --- | --- |
| BIA data | |  |
| Total body water, L | 39.10 (34.12-42.12) |  |
| ECW, L | 15.7 (14.1-17.7) |  |
| TBW, L | 39 (34-43) |  |
| ECW/TBW | 0.405 (0.39-0.41) |  |
| Skeletal muscle mass, kg | 28.05 (24.30-30.77) |  |
| Segmental lean, right arm, kg | 2.47 (2.23-2.88) |  |
| Segmental lean, left arm, kg | 2.415 (2.11-2.91) |  |
| Segmental lean, right leg, kg | 8.74 (7.55-10.53) |  |
| Segmental lean, left leg, kg | 8.50 (7.61-10.07) |  |
| Thigh thickness on ultrasound |  |  |
| Right thigh muscle thickness, cm | | 1.24 (0.85-1.32) |
| Left thigh muscle thickness, cm | | 1.14 (0.88-1.38) |
| Average thigh muscle thickness, cm | | 1.11 (0.87-1.35) |

Values are expressed as median (interquartile range). BIA: bioelectrical impedance analysis; ECW: extracellular water; TBW: total body water.
